# Use of Multimodality Imaging in the Evaluation of Patients With Spondyloarthropathies and Sacroiliitis

**DOI:** 10.7759/cureus.57185

**Published:** 2024-03-29

**Authors:** Mahi Basra, Hemangi Patel, Alexandria Sobczak, Jordan Ditchek, Alejandro Biglione, Marc M Kesselman, Alessandra Posey

**Affiliations:** 1 Osteopathic Medicine, Nova Southeastern University Dr. Kiran C. Patel College of Osteopathic Medicine, Clearwater, USA; 2 Sports Medicine, Nova Southeastern University Dr. Kiran C. Patel College of Osteopathic Medicine, Fort Lauderdale, USA; 3 Osteopathic Medicine, Nova Southeastern University Dr. Kiran C. Patel College of Osteopathic Medicine, Fort Lauderdale, USA; 4 Radiology, Nova Southeastern University Dr. Kiran C. Patel College of Allopathic Medicine, Davie, USA; 5 Internal Medicine, Wellington Regional Medical Center, Wellington, USA; 6 Rheumatology, Nova Southeastern University Dr. Kiran C. Patel College of Osteopathic Medicine, Davie, USA; 7 Sports Medicine, Nova Southeastern University Dr. Kiran C. Patel College of Osteopathic Medicine, Davie, USA

**Keywords:** psoriatic arthritis, ankylosing spondylitis, multimodality imaging, spondyloarthropathy, sacroiliitis

## Abstract

Spondyloarthropathy (SpA) is one of the most common causes of low back pain. It is caused by inflammatory arthritis in the spine, manifesting in various forms such as psoriatic arthritis (PsA), ankylosing spondylitis (AS), and sacroiliitis. A comprehensive systematic literature search was done to evaluate and compare MRI, CT, single-photon emission CT, PET, ultrasound (US) imaging, low-dose CT, and diffusion-weighted imaging (DWI) techniques in assessing SpAs. The search strategy was constructed by an analysis of key terms from relevant articles in MEDLINE ProQuest, Embase, and PubMed. The key terms used to search for these articles were “SpA,” “sacroiliitis,” “spondylitis,” “psoriatic arthritis,” “MRI,” “CT scan,” “x-ray,” “magnetic resonance imaging,” “computed tomography,” “bone density,” and “ultrasound.” A total of 1,131 articles published in English between January 1, 2003, and October 15, 2023 were identified and screened for eligibility by members of the research team, which resulted in 69 total articles selected for the final review. US has played an important role in visualizing joint inflammation and enthesitis (inflammation of the enthesis), which are common features of PsA. Although MRI and CT are considered more reliable modalities for diagnosing active sacroiliitis, US imaging with Doppler flow can also be useful in conjunction with CT images to visualize abnormal blood flow in the sacroiliac joints, as well as other joints affected by inflammatory arthritis. MRI provides increased diagnostic confidence in the diagnosis of sacroiliitis in active AS patients when compared to CT. CT is more sensitive than plain radiographs. The PET activity score showed a good correlation in diagnosing inflammatory sacroiliitis but lacked in identifying structural lesions. CT has high diagnostic accuracy, but it exposes patients to a high radiation dose. MRI visualizes joint and tissue inflammation, bone, and bone marrow change and can identify peripheral inflammation in soft tissue and joints in patients diagnosed with PsA. MRI can also visualize bone marrow changes and subchondral edema, which can aid in the early diagnosis of ankylosing SpA and gauge disease severity. DWI and short-tau inversion recovery imaging are both MRI techniques used in detecting sacroiliitis. MRI and CT are shown to be reliable imaging modalities for the diagnosis of sacroiliitis; however, it was found that Doppler US played an accurate role in the diagnosis as well. MRI visualizes joints and tissue with the most precision, making it useful in evaluating patients with PsA, while PET CT is useful in the diagnosis of inflammatory sacroiliitis patients. There is limited literature available comparing the multiple modalities of imaging available for each SpA. The review’s objective is to analyze imaging findings in patients diagnosed with sacroiliitis and SpAs. The findings in this literature review are valuable for properly assessing and diagnosing patients suffering from SpAs.

## Introduction and background

Spondyloarthropathies (SpAs) are a group of seronegative and inflammatory arthritis that typically involve the axial spine. This chronic rheumatic disease starts before the age of 40 and may also spread to involve peripheral joints causing arthritis, entheses causing enthesitis, digits causing dactylitis, and the axial spine typically causing sacroiliitis [1]. Subtypes of SpAs include ankylosing spondylitis (AS), enteropathic SpAs, including Crohn’s disease and ulcerative colitis, reactive arthritis, psoriatic arthritis (PsA), and undifferentiated SpA [2]. The spA is further differentiated into axial SpA (axSpA), in which sacroiliitis symptoms predominate, or peripheral SpA, in which arthritis, enthesitis, or dactylitis symptoms predominate. Laboratory findings in AS typically include a positive Human Leukocyte Antigen B27 (HLA-B27), which is part of a specific gene locus in the major histocompatibility complex class I of genes on chromosome 6. The presence of the HLA B27 allele is strongly associated with the development of SpAs [1]. The absence of a rheumatoid factor, also typical in SpA, helps to differentiate the disease from rheumatoid arthritis [1]. The Assessment of Spondyloarthritis International Society (ASAS) classification criteria are now widely accepted for the diagnosis of axSpA, which requires either (1) sacroiliitis on imaging with greater than one SpA feature (inflammatory back pain (IBP), arthritis, enthesitis, uveitis, dactylitis, psoriasis, Crohn’s disease/ulcerative colitis, a good response to non-steroidal anti-inflammatory drugs, a family history of SpA, HLA-B27 positive, or elevated CRP levels) or (2) HLA-B27 positive with greater than two SpA features [3].

PsA is a type of SpA that is accompanied by skin and nail changes. Psoriasis is a skin disease that affects the musculoskeletal system, involving the tendon sheaths, bursae, and adjacent bone and bone marrow of the affected joint regions. PsA affects the proximal and distal joints and differs from rheumatoid arthritis by (a) generally lacking metacarpophalangeal (MCP) joint involvement, (b) asymmetrical joint involvement, and (c) gender preference. Specifically, for PsA, there is asymmetric distribution, distal interphalangeal joint (DIP) joint involvement, bone erosions present, ankylosis, and resorption of distal phalanges. There are multiple imaging modalities used for PsA, with ultrasound (US) and MRI showing many changes that can be detected earlier than with conventional radiography. MRI is able to show bone erosion and bone marrow edema that can indicate future joint damage [4].

AS characteristically presents with inflammation of the sacroiliac joint (SIJ), which may occasionally spread to other regions of the spine as spondylitis, spondylodiscitis, or spondyloarthritis. Associated symptoms in patients may include uveitis, psoriasis, inflammatory bowel disease, aortitis, amyloidosis, or osteopenia. Ultimately, as the disease progresses, fusion of the axial joints occurs, causing significant morbidity in patients [5]. The predominant symptom present in patients is typically sacroiliitis-induced IBP [5]. The New York (NY) AS Diagnostic criteria were initially used in 1966 and then modified in 1984, which require the presence of sacroiliitis on plain radiographs for AS diagnosis [3]. Grade 0 is classified as normal; grade 1 is classified as blurring of joint space margins; grade 2 can show some small areas of erosion or localized sclerosis without alteration of joint width; grade 3 shows moderate to advanced sacroiliitis with sclerosis, erosions, and widening, narrowing, or partial ankylosis; and grade 4 must show complete ankylosis [6].

Although sacroiliitis is typically one of the first manifestations of AS, the spine may also show small erosions on plain radiographs with early spondylitis. A “bamboo spine” appearance caused by the osseous bony outgrowths of the spine called syndesmophytes may also be seen. Additionally, bilateral and symmetric hip involvement with uniform joint space narrowing is common. Other findings, such as uniform knee joint space narrowing and marginal periostitis asymmetrically in the hands, may be seen on plain radiographs [6].

## Review

Methods

Search Strategy and Selection Criteria 

A comprehensive literature search was performed on October 16, 2023 using Embase, MEDLINE ProQuest, and PubMed databases. This systematic review utilized the Preferred Reporting Items for Systematic Reviews and Meta-Analyses (PRISMA) statement by the Cochrane Collaboration. Either clinical trials, cohort studies, cross-sectional studies, retrospective studies, or prospective studies must have been conducted in the human patient population between January 1, 2003 and October 1, 2023 to be included. Review articles, pathology not pertaining to SpAs, and non-English articles were excluded. Additionally, studies that did not pertain to radiographic imaging were excluded. These criteria were selected and adhered to in order to complete the objectives of this review.

Key Terms

The key terms used to search for articles were spondyloarthropathy, sacroiliitis, spondylitis, psoriatic arthritis, MRI, CT scan, scan, x-ray, XR, xray, magnetic resonance imaging, computed tomography, bone density, and ultrasound. Databases were searched using the Boolean operators “AND” and “OR” as follows: ((“Spondyloarthropathy*” OR (“Sacroiliitis*” OR “spondylitis*” OR “psoriatic arthritis*”)) AND (“MRI*” OR “CT Scan*” OR “Scan*” OR “X Ray*“ OR “XR*” OR “Xray*“ OR “Magnetic Resonance Imaging*” OR “Computed Tomography*“ OR “Bone Density*” OR “Ultrasound*“)). These key terms were selected based on the current literature available on the diagnosis of SpAs.

Evaluation Process 

**Figure 1:**
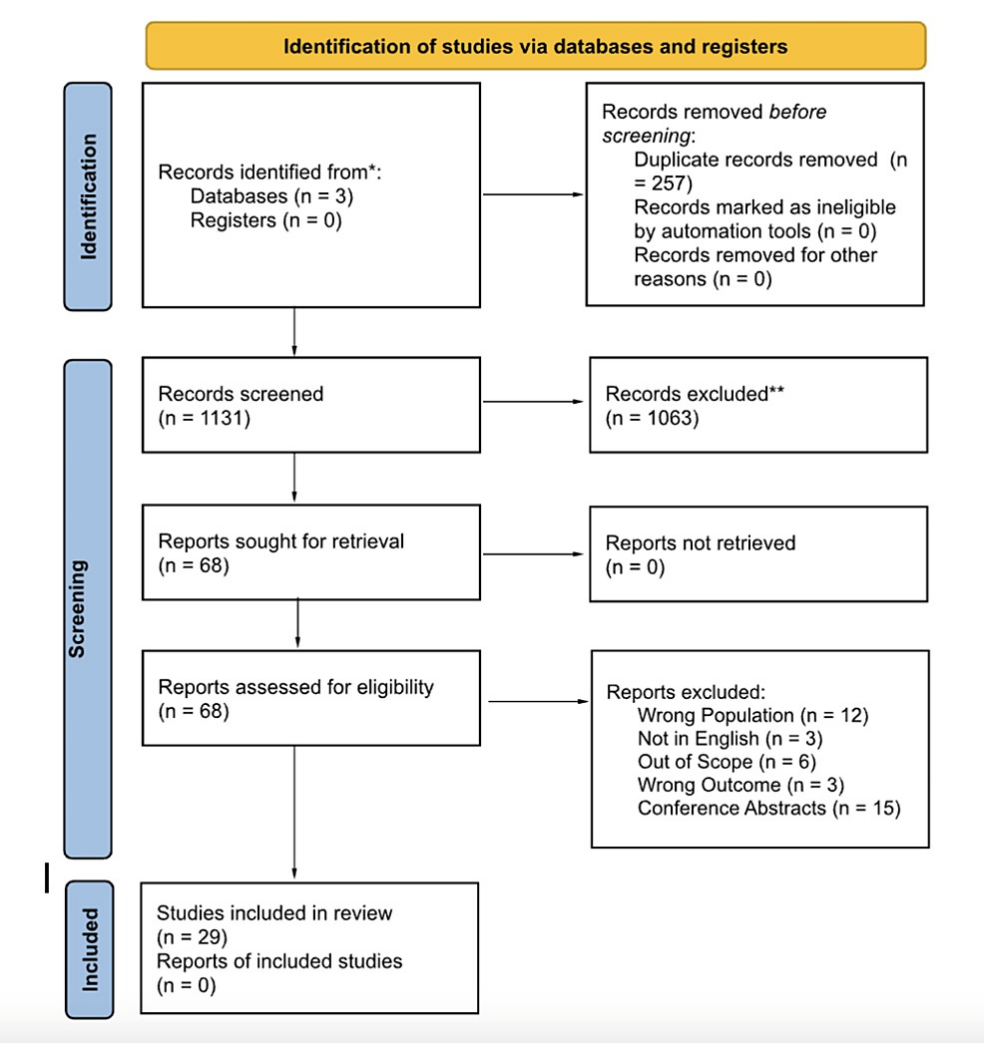
PRISMA diagram PRISMA, Preferred Reporting Items for Systematic Reviews and Meta-Analyses

The process of inclusion for the articles was portrayed in the PRISMA diagram (Figure 1). The authors (MB, HP, and AS) evaluated the same 1,131 articles after 257 duplicate articles were removed. Based on further evaluation and utilization of the inclusion criteria, 1,063 articles were excluded. A total of 68 articles were sought for retrieval and assessed for eligibility. Authors (MB, HP, and AS) independently reviewed each full-text publication. Once further analysis was conducted, 11 articles were excluded due to an incorrect patient population defined as patients without SpA diagnoses, three articles were not in English, six articles were out of scope, six articles had an incorrect outcome, and 15 articles were solely conference abstracts without full-text publication. All authors discussed disagreements and resolved conflicts. A total of 29 articles were included in the systematic review.

Data Collection

All authors (MB, HP, and AS) independently evaluated each full-text article. This information was then utilized to develop a data abstraction table including references, study design, sample size and methods, results, and limitations of the study, as seen in Table 1. After synthesis and qualitative analysis of the data were performed, the narrative review was written.

**Table 1:** Results of all the studies included 2D, two-dimensional; 3D, three-dimensional; ADC, apparent diffusion coefficient; AS, ankylosing spondylitis; ASAS, Assessment of SpondyloArthritis International Society; BASDAI, Bath Ankylosing Spondylitis Disease Activity Index; CDUS, color Doppler US; CIL, corner inflammatory lesion; DAPSA, Disease Activity Index for Psoriatic Arthritis; DWI, diffusion-weighted imaging; ESR, erythrocyte sedimentation rate; FS, fat-suppressed; GSS, gray-scale synovitis; IBP, inflammatory back pain; LSTV, lumbosacral transitional vertebrae; NY, New York; PD, power Doppler; PsA, psoriatic arthritis; SIJ, sacroiliac joint; SpA, spondyloarthropathy; SPECT, single-photon emission CT; STIR, short-tau inversion recovery; US, ultrasound; WB-MRI, whole-body MRI

Reference	Study design	Data collection	Study aim	Findings	Limitations
Parghane et al. (2017) [2]	Prospective study	N = 155 patients. Clinical evaluation using BASDAI to measure ESR, CRP, planar 99mTc-Methylene scintigraphy, SPECT/CT, and MRI of the pelvic region. On planar bone scintigraphy and SPECT, a score of 0, 1, or 2 was assigned if tracer uptake in SIJ was noted; a score of 2 was considered positive for sacroiliitis diagnosis	Compare the role of 99mTc-Methylene SPECT/CT in the detection of sacroiliitis with MRI	99mTc-Methylene SPECT/CT had comparable diagnostic accuracy to MRI regarding specificity and sensitivity. 99mTc-Methylene SPECT/CT showed better accuracy than planar bone scintigraphy, ESR, CRP, and BASDAI scoring	Nondiagnostic CT was done as part of the SPECT/CT study, so the incremental value of CT changes was not assessed separately
Ghanem et al. (2007) [4]	Cross-sectional study	N = 25 patients with PsA were diagnosed by an experienced rheumatologist who had no systemic therapy before evaluation on MRI and was seronegative for rheumatoid factors. All 25 participants underwent MRI to evaluate the imaging findings of PsA	MRI findings of patients with a clinical diagnosis of PsA	A total of 88% of patients had soft tissue edema identified on MRI, 92% of patients had joint effusions visualized on MRI, and 84% of patients had bone marrow edema. All patients had contrast enhancement	Low sample size
Strobel et al. (2010) [5]	Retrospective clinical trial	A total of 15 patients with AS and active disease, 13 with mechanical low back pain, both evaluated with whole-body (18)F-fluoride PET/CT	Evaluate the performance of (18)F-fluoride-PET/CT (PET/CT) for the diagnosis of SIJ arthritis in patients with active AS	(18)F-fluoride-PET/CT (PET/CT) may play a role in the diagnosis of sacroiliitis in active AS and can be used in the replacement of bone scintigraphy when molybdenum is in shortage	None stated
Nadeem et al. (2022) [8]	Retrospective study	A total of 54 patients undergo initial SIJ MRI and then subsequent CT with 14.4 weeks in between the two imaging modalities	Determine if CT could improve the diagnosis of SIJ erosions in patients with equivocal MRI findings	CT increased diagnostic confidence and reliability in detecting SIJ erosions in comparison to MRI, so CT can detect SIJ erosions in patients with equivocal MRI findings	Rheumatology referred patients, so findings may not transfer to routinely referred patients. MRI and CT were evaluated in isolation for the presence or absence of erosions. Patients may have developed SIJ erosions between the initial SIJ MRI and subsequent SIJ CT. The MRI protocol did not have volumetric MR imaging to improve the overall diagnostic confidence of erosions
Devauchelle-Pensec et al. (2012) [9]	Prospective cohort study	N = 489 patients with suspected SpA. Participants underwent imaging to assess the performance of CT in detecting spondyloarthritis	To assess the usefulness of CT in identifying sacroiliitis	CT scanning facilitates the diagnosis of AS better than radiography	Not all patients had CT. Scanning cannot evaluate the usefulness of patients who did not have buttock pain for more than six months or when sacroiliitis was not present on radiographs
Hoenen-Calvert et al. (2007) [10]	Retrospective clinical trial	N = 32 patients. FS-T2 and FS-T1 images were obtained in either axial or peripheral symptomatic regions. Images were blindly scored and evaluated for reproducibility and frequency	To determine the most valuable MRI criteria for diagnosing SpA with axial or peripheral involvement	Synovitis, ligament inflammation, bone marrow edema, and tenosynovitis were most prevalent in peripheral regions. Peripheral fat inflation, chondral lesions, enthesophytes, fusion, and erosions were most prevalent in axial regions. Erosions and enthesophytes were structurally present. No inflammatory axial criteria had good interobserver reproducibility	Small sample size
Chan et al. (2018) [11]	Retrospective study	N = 305 patients with chronic back pain. STIR and DWI sequences were obtained	To compare DWI imaging with STIR sequence in SpA diagnosis	DWI had similar sensitivity and specificity to the STIR sequence. The STIR sequence had better reliability. DWI and STIR sequences were comparable in detecting active sacroiliitis	Cross-sectional design
Chan et al. (2020) [12]	Retrospective study	N = 369 patients with a known axSpA diagnosis, with 117 patients in the control group. CILs locations were assessed via the spine and SIJ MRI	To determine whether the location of CILs would affect the diagnosis of axSpA in MRI	CIL in the thoracic spinal segments were the most frequent and accounted for 74% of the total number of CILs	Patients had long-standing diseases rather than newly diagnosed
Inanc et al. (2005) [13]	Comparative study	N = 54 patients with IBP fulfilling European SpA criteria; scintigraphy scanning was done in 80 healthy patients to establish control. Patients were divided into two groups according to changes on plain radiography. Quantitative SI scintigraphy and MRI were performed	Analyze various diagnostic imaging techniques in SpA patients with inflammatory low back pain	Sensitivity was 61%, 55%, and 89% of plain radiographs, SI scintigraphy, and MRI, respectively. MRI was the most sensitive method of detecting both acute and chronic changes in SpA patients	A low sample size. Comparison with CT imaging was not done
Gupta et al. (2006) [14]	Cross-sectional validation study	N = 104 patients with suspected axial spondyloarthritis. A 3D coronal oblique reconstruction parallel to the sacrum’s long axis was created and compared to 2D techniques for sacroiliitis diagnosis	To compare the diagnostic efficacy of 3D vs 2D imaging for sacroiliitis	The 3D imaging had a 10% greater sensitivity for detecting inflammatory changes and a 15% greater sensitivity for detecting mechanical changes. The 2D imaging had an 11% greater specificity for detecting active sacroiliitis and was better at detecting bony ankylosis	******
Bredella et al. (2006) [15]	Retrospective clinical trial	N = 18 patients with moderate-severe AS. T1-weighted, (FS)-T2, STIR, and fat-saturated contrast-enhanced T1-weighted sequences were evaluated	To determine whether MRI findings of SIJs are able to separate active vs. inactive disease in patients with AS	A total of 17 patients had abnormal SIJ findings. Ten patients had active disease on MRI, displaying abnormal enhancement and subchondral bone marrow edema. Fourteen patients had fatty subchondral bone marrow. No correlation between disease activity or duration	There is a small sample size and the absence of pathologic tissue to confirm disease activity. Additionally, reviewers were aware of the patient's diagnosis
Raynal et al. (2019) [16]	Prospective study	N = 23 patients with active SpA. Pelvic radiographs, SIJ MRI, and 18-FNa PET/CT were performed and compared in the same month and quantitatively assessed	To assess increased SIJ uptake on 18-FNa PET/CT and compare with MRI SIJ assessment	Inflammation on STIR MRI sequences can be detected earlier. Activity was better reflected by bone formation using the 18-FNa radiotracer due to osteoblast activity. The 18-FNa uptake was associated with inflammation, not structural change. A total of 52.2% of patients presented with inflammation; 10 patients had inflammatory lesions on MRI. No correlation was found between positive PET/CT and inflammatory sacroiliitis on MRI	Small sample size. Absence of a control group
Weber et al. (2010) [17]	Cross-sectional study	N = 187 patients: 75 patients with AS, 27 with pre-radiographic IBP, 26 with nonspecific back pain, and 59 healthy controls. After images were taken, bone marrow edema, fatty infiltration, ankylosis, and erosion were recorded	Differentiate patients with SpA from patients with nonspecific back pain using MRI of SIJs	MRI has excellent diagnostic utility for SIJ. Bone marrow edema is the most common inflammatory lesion in patients with IBP. Erosions and fat infiltrations were the most common inflammatory lesions in patients with AS	Small sample size. Cross-sectional design. Low number of patients with IBP
Carvajal et al. (2020) [18]	Cohort study	N = 688 patients with IBP for greater than three months, but less than three years. Pelvic radiographs were assessed by two blinded readers for the classification of LSTVs	To assess the potential impact of LSTVs on SIJ and assess prevalence in accordance with sacroiliitis on plain radiographs and MRI	LSTVs were found in 29.1% of patients: Castellvi Ia in 7.8% of patients, Ib in 11% of patients, IIa in 2.9% of patients, IIb in 1.7% of patients, IIIa in 1% of patients, IIIb in 3% of patients, and IV in 1.4% of patients. There was an 8% greater number of patients with LSTV meeting NY criteria for radiographic sacroiliitis and a 10% greater number of patients with LSTV meeting ASAS criteria for sacroiliitis	The group size for each subtype of LSTV was small despite the large sample size. No comparison was done with healthy individuals (control group)
Giraudo et al. (2016) [19]	Prospective study	N = 101 patients with suggestive clinical findings of SpA. A total of 105 patients underwent the paT2 protocol, and 41 patients underwent paPD-FS in comparison to the gold standard post-contrast sequence	To compare para-axial T2w-TSE (paT2) and FS proton density (paPD-FS) diagnostic utility on MRI	The paT2 sequence was used for chronic findings due to the lack of fat suppression. The paT2 sequence provides less information than fat suppressed sequences for the evaluation of acute changes. The paPD-FS sequences have a high signal-to-noise ratio when compared to other fat suppression techniques and have 100% sensitivity and specificity for enthesitis, synovitis, and ankylosis. The inclusion of the para-axial plane would be beneficial in evaluating the complicated SIJ anatomy	Small sample size. Only 41 patients underwent the paPD-FS protocol. No correlation between imaging and histology
Weber et al. (2010) [20]	Comparative primary study	N = 32 patients with SpA were scanned by MRI	Compare WB-MRI vs. conventional MRI in assessing active spine lesions	WB-MRI and conventional MRI had high reliability for diagnosing inflammatory spine lesions in patients with SpA	Low sample size
Poggenborg et al. (2015) [21]	Pilot study	N = 48 patients total. PsA in 18 individuals, axSpA in 18 individuals, and HSs in 12 individuals. Patients with PsA were diagnosed using Moll and Wright criteria, or European SpA study group criteria. All participants underwent WB-MRI to visualize inflammatory and structural lesions associated with PsA	Test the ability of WB-MRI to depict the central/axial and peripheral manifestations of PsA and SpA	Readability and reproducibility were low in peripheral joints but high in more proximal joints such as the hip and knee. PsA individuals were shown to have similar readings between the hands and feet, whereas SpA patients had a higher percentage of lesions identified in the feet than the hands. Synovitis was more often seen on WB-MRI than bone marrow edema or erosion	Low sample size. More advanced definitions of scoring and reader training advised
Weber et al. (2015) [22]	Cohort study	N = 130 patients greater than 50 years old with back pain, with 20 controls. Clinical examination, SIJ, and spine MRI were performed, and differences were analyzed	Assess the additive diagnostic value of spine MRI both separately and combined with SIJ MRI in nr-axSpA when compared to SIJ MRI alone	Combined spine and SIJ MRI did not add much diagnostic value to diagnosing nr-axSpA	Potential false positives or negatives based on clinical assessments
Son et al. (2020) [23]	Retrospective cohort study	Sixty-eight patients who underwent 18F-NaF PET/CT imaging between April 2015 and April 2017	Examination of fluorine-18 sodium fluoride (18F-NaF) PET/CT (PET/CT) findings in patients with inflammatory low back pain and evaluation of the use of 18F-NaF PET/CT in diagnosing AS according to the ASAS criteria	The 18F-NaF PET/CT is useful in diagnosing AS	Small sample size
Husic et al. (2014) [24]	Prospective study	N = 70 patients with PsA based on the classification for PsA who also had peripheral articular manifestations of the disease. All individuals underwent US to compare clinical symptoms and lesions found on US	Look at the association between clinical composite scores of PsAA and US pathology	B-mode and PD synovitis were positively correlated with DAPSA scores. ESR is positively correlated with GSS and PD joint scores. A total of 67.1% of patients had entheseal erosions, and all patients had at least one enthesophyte on US	Low sample size. Low reliability of GSS. Extensive US assessment
Bozgeyik et al. (2008) [25]	Cross-sectional study	N = 65 patients, a total of 20 patients with AS, 20 patients with low back pain, and 25 health controls. All participants underwent DWI to assess its role in identifying AS	Investigate the role of DWI in identifying early AS	DWI provides a fast alternative to contrast-mediated MRI. Subchondral marrow ADC levels distinguished active AS from control groups	Small sample size
Sanal et al. (2013) [26]	Cross-sectional study	A total of 21 patients with AS were examined with DTIR, FST1/Gd, and DWI sequences. Patients underwent contrast, STIR, and DWI imaging to assess the contrast-to-noise ratio	Compare noise ratios of the SIJ between different sequences of MRI in patients with AS	A significant difference in noise ratio was found between STIR and other MRI sequences, but there was no difference between contrast MRI and DWI	Small sample size
Lee et al. (2016) [27]	Retrospective study	N = 53 patients with AS. All participants underwent MRI imaging of the spine and facet joints	Investigate the reliability of MRI for identifying inflammation in the facet joints	The MRI had a high ICC value of 85.7%. AS could be identified on MRI by visualizing spinal inflammation. ASAfacet scores were related to ESR levels	Small sample size
Baraliakos et al. (2005) [28]	Cross-sectional study	N = 38 participants with active AS were evaluated on MRI	Compare T1-weighted, fat-saturated, spin echo after contrast (Gd-DTPA) with STIR sequences	The interclass correlation coefficients were 0.91 and 0.86. High reliability was found when reviewing the entire spine versus lower reliability when looking at individual regions (cervical, thoracic, and lumbar)	Small sample size
Weber et al. (2009) [29]	Cross-sectional study	N = 95 total participants: 35 patients with AS using NY criteria, 25 with IBP, and 35 controls. Patients underwent different types of MRI imaging to assess how well different modalities can visualize lesions associated with AS	Investigate the diagnostic utility of WB-MRI in identifying AS	Two or more CILs were diagnostic for AS when using WB-MRI. Sensitivity and specificity were 69% and 94%	Small sample size
Hamdi et al. (2013) [30]	Prospective study	N = 60 patients with AS defined using modified NY criteria for AS	Investigate the use of US in identifying erosion, swelling, and new bone formation in peripheral entheses compared to plain radiographs	The US was quite sensitive in identifying erosions, bone formation, and swelling, but lacked specificity when compared with radiographs	Low sample size
Mohammadi et al. (2013) [31]	Cross-sectional study	N = 81 participants were included in this study. There were 30 controls and 51 with a clinical diagnosis of AS according to the Modified NY criteria. Patients underwent contrast-enhanced MRI and CDUS of the SIJs, and vascularization was assessed	Investigate CDUS as a diagnostic tool for sacroiliitis in patients with AS	CDUS was able to predict sacroiliitis with 82% sensitivity and 92% specificity. Of the patients with active AS, 81.5% of them had a pulsatile monophasic flow on CDUS, and 18.5% had no flow	One radiologist performed all ultrasonographic evaluations. ESR was not measured in the control group
Hu et al. (2015) [32]	Preliminary study	N = 114 total participants, with 84 individuals with AS and 30 controls. Participants underwent fused CT/US imaging to visualize blood flow in the SIJs	Investigate if fusion CT-US is useful in identifying inflammation and abnormal vascularization in the SIJs	CT and US images can be fused to more accurately identify areas of abnormal vascularization in the SIJ	Unable to compare US-CT to US-MRI. Unable to visualize the fourth or fifth level because the sacral foramina were too small to distinguish on US
Lee et al. (2007) [33]	Cross-sectional study	N = 37 patients with determined or probable AS; AS was diagnosed based on modified NY criteria. All participants underwent CT imaging to assess SpA changes in the SIJs	Investigate the ability of CT to detect early-stage AS	CT-graded early-stage sacroiliitis is higher than in plain radiographs. Radiographic criteria for sacroiliitis were met in more patients using CT than plain radiographs	Low sample size

Results

The diagnosis of AS is made when there is one clinical criterion plus a grade 2 bilateral or grade 3 unilateral finding on plain radiographs, known as the NY criteria [5]. The clinical criteria of the NY criteria include low back pain and stiffness for greater than three months improving with exercise and/or limited range of motion of the lumbar spine in lateral bending and forward bending and/or limited chest expansion in relation to normal values based on age and sex [7]. However, because radiographic changes occur in the later stages of AS, the classification based on the NY criteria is not used to determine the early stages of the disease [2]. The early stages of the disease include SIJ and spinal inflammation, which is not present on radiographs, while the late stages of the disease include inflammatory and structural changes, which can be seen on radiographs [8]. Although conventional radiography is the imaging technique of choice for diagnosing SpAs, the oblique and curved contour of the SIJ makes conventional radiography difficult when detecting anatomic changes such as erosions, sclerosis, bony bridges, and ankylosis [9]. Aside from CT scans, there are many other imaging modalities used for diagnosis, including PET, MRI, and 99mTc scintigraphy, which will be further discussed.

Sacroiliitis

MRI: Although classification and diagnosis are based on plain radiographic evidence based on the aforementioned NY criteria, MRI may allow for earlier detection of disease. A plain radiographic diagnosis may require between five and seven years of structural damage, while an MRI can reveal acute inflammatory changes before structural lesions occur. An MRI examination for patients focuses on either the axial or peripheral region, whichever is most symptomatic. Hoenen-Clavert et al. evaluated 32 patients with fat-suppressed (FS)-T2 and (FS)-T1 images to determine the most valuable diagnostic criteria. Synovitis, ligament inflammation, bone marrow edema, and tenosynovitis were most prevalent in peripheral regions, while peripheral fat inflammation, chondral lesions, enthesophytes, fusion, and erosions were most prevalent in axial regions. Erosions and enthesophytes were structurally present. Inflammatory axial criteria did not have good interobserver reproducibility [10]. Diffusion-weighted imaging (DWI) has been investigated as a potential early diagnostic method for sacroiliitis in axSpA patients. This technique quantifies the diffusion properties of water molecules with a specific structure. However, due to its low spatial resolution, it has not been more beneficial than short-tau inversion recovery (STIR) sequences. Chan et al. evaluated the diagnostic utility of DWI versus STIR sequence in the early diagnosis of sacroiliitis in axSpA. Results showed that DWI had similar sensitivity and specificity to the STIR sequence and were both comparable in detecting active sacroiliitis. However, the STIR sequence had better reliability in diagnosing active sacroiliitis. This may be due to DWI’s poor visuospatial resolution [11]. Furthermore, corner inflammatory lesions (CILs) are seen as an increased STIR sequence at a vertebral body’s corner. This typically remains normal or hypodense in T1-weighted sequences. More than three CILs have been suggested to have a positive spinal MRI diagnosis for axSpA by the ASAS. Chan et al. investigated the diagnostic utility of CILs in the diagnosis of axSpA. In a group of 309 patients, CILs in the thoracic spinal segments were the most frequent and accounted for 74% of the total number of CILs. Thoracic CILs were more common in axSpA individuals when compared to the control group (presenting with nonspecific back pain). MRI for sacroiliitis and greater than five CILs were comparable for specificity when the whole spine was looked at [12].

MRI has been noted as the most sensitive method of detecting both acute and chronic changes in SpA patients when compared to plain radiographs and SI scintigraphy. Inanc et al. found that sensitivity was 61%, 55%, and 89% of plain radiographs, SI scintigraphy, and MRI, respectively [13]. With the comparison of three-dimensional (3D) isotropic imaging, two-dimensional (2D) multisequence MRI imaging had an 11% greater specificity for detecting active sacroiliitis and was better at detecting bony ankylosis in patients with suspected axial spondyloarthritis [14]. Additionally, Bredella et al. evaluated 18 patients and found that MRI was sensitive in depicting sacroiliitis because inflammatory edema lesions with high water content were able to be differentiated from lesions with fibrotic tissue and sclerosis due to lower water content. Sacroiliitis may result from inflammation from local fat metabolism, leading to visible subchondral edematous areas visible on MRI [15]. Raynal et al. found a significant correlation between sodium fluoride labeled with fluorine 18 (18-F-NaF) PET/CT activity and inflammation score on MRI. This is correlated with osteoblastic activity on 18-F-NaF PET/CT and bone marrow edema on MRI. The combination of both bone marrow edema and the deposition of fat had the highest uptake [16].

It is evident that MRI has great diagnostic utility in evaluating the SIJ and can be used to evaluate structural changes. These changes can occur as early as less than 24 months after symptom onset in patients. Although bone marrow edema and fat infiltration have been used to characterize SpA in MRI, Weber et al. found that these changes are nonspecific and may occur in healthy individuals. Both bone marrow edema and erosions were noted by more than two readers in 24% of healthy controls and 27% of patients with nonspecific back pain. Weber et al. hypothesize that this may be due to signal alterations or degenerative changes. This study found that erosions had an 81% sensitivity for SpA and can be used to improve diagnostic validity in MR sequences [17].

However, as with all imaging, limitations must be taken into account with regard to anatomical variations in patients. Alegria et al. assessed the influence of lumbosacral transitional vertebrae (LSTVs) on SIJs. LSTVs are anatomical variations of the lumbar spine that can be seen on plain radiographs. Due to the lack of specificity of ASAS criteria, patients without axSpA or IBP can still meet sacroiliitis ASAS MRI criteria. Thus, patients with bone marrow edema on MRI may still meet the criteria for sacroiliitis, but it may be due to other reasons, such as in healthy women after childbirth. This study found that there was an 8% greater number of patients with LSTVs meeting NY criteria for radiographic sacroiliitis and a 10% greater number of patients with LSTVs meeting ASAS criteria for sacroiliitis. Thus, further studies are needed in healthy individuals to truly assess the impact of LSTVs on sacroiliitis diagnosis [18]. Additionally, due to the complex anatomy of the SIJ, a para-axial plane may be required to accurately assess the anatomy and ligaments of the SIJ. Giraudo et al. examined the para-axial T2-weighted TSE sequence (paT2) for chronic findings and the FS proton density (paPD-FS) sequence for assessing chronic and acute changes. They utilized post-contrast images as the gold standard, which served as a reference. A comparison was only performed between paT2 and paPD-FS, with the paracoronal T1-weighted TSE sequence (pcT1) as the gold standard. paPD-FS had 98.9% sensitivity and 99.1% specificity in identifying one quadrant with bone marrow edema in the SIJ. This sequence was used to identify both acute and chronic changes due to its fluid-sensitive property and high signal-noise ratio when compared to STIR. Although this was only performed in 41 patients out of 101 in this study, it had a 100% sensitivity and specificity for ankylosis, synovitis, and enthesitis. Both paT2 and paPD-FS aided in the accurate assessment of the intricate anatomy of the SIJ. Thus, the authors suggest paPD-FS could potentially replace post-contrast scans and increase diagnostic capability in spondyloarthritis [19].

Whole-body MRI (WB-MRI) and conventional MRI both have excellent reliability for diagnosing inflammatory spine lesions in patients with SpA [20]. WB-MRI allows for the assessment of all axial and peripheral joints. Both SpA patients and PsA patients have increased bone marrow edema on WB-MRIs. SpA patients had the most SIJ involvement in terms of inflammatory and structural changes, while PsA patients had peripheral joint involvement [21]. However, there was little to no diagnostic value in diagnosing nr-axSpA when utilizing both spine and SIJ MRI compared to SIJ MRI alone [22].

PET scan and CT: MRI has been superior to other imaging techniques used for AS because it has greater accuracy in detecting early cartilaginous changes and bony erosions [2]. However, PET scans have the potential to be the most sensitive in relation to MRI and CT for detecting early bone remodeling before AS changes begin, as there is a significant correlation found for AS, SIJ, and sacrum ratio using the PET activity score [16]. This correlation is not as apparent when comparing AS and the inflammation score on an MRI. Further, when compared to CT scans, PET scans can detect earlier bone remodeling and structural lesions than CT scans due to the higher uptake of PET scans in the SIJ region [16]. The 18F-NaF PET/CT has greater sensitivity than MRI or CT scans for detecting inflammatory and/or structural sacroiliitis changes by being able to detect early lesions, changes, or scars that CT scans or MRIs are unable to detect [16]. Additionally, 18F-NaF PET/CT can also detect sacroiliitis in non-radiographic SpA patients, which MRI or CT cannot [16,23]. The study conducted by Raynal et al. included 23 patients with active SpA who had radiographs, MRIs, CT scans, and PET scans performed. The findings were consistent with 20/23 having a positive PET scan, while only 7/23 had radiographs, 10/23 CT scans, and 10/23 MRIs with positive findings of inflammatory sacroiliitis. In patients with grade 3 sacroiliitis, increased uptake was found in PET scans, which may correlate directly to the post-inflammatory repair state and cannot be found on MRI or CT scans. This finding exemplifies that the sensitivity of PET scans is greatest in relation to stand-alone CT scans and MRIs.

Further, conventional scintigraphy with bone-seeking tracers such as 99mTc-labeled diphosphonates has been used in patients with AS for a while but includes the need for molybdenum [5]. There has been a worldwide shortage of molybdenum, which makes the use of conventional scintigraphy difficult. Thus, PET scans have been used more frequently for diagnosing sacroiliitis in AS patients with active disease. Conventional scintigraphy has a sensitivity of 52%, while 18F-fluoride PET/CT was found to have a sensitivity of 80% [5]. The PET tracer has greater bone uptake and rapid blood clearance, leading to a superior lesion-to-background ratio in relation to 99mTc-labeled phosphonates, as well as a superior gamma camera, increasing spatial resolution and sensitivity [5]. Additionally, the CT aspect of PET/CT allows for more precise localization of the lesion and greater visualization of chronic changes, including but not limited to erosions, sclerosis, and ankylosis. Thus, the sensitivity of PET/CT is greater for detecting sacroiliitis in AS as well as in early SpA in relation to conventional scintigraphy.

Further, when there are contraindications to MRI use in patients, an alternative technique for diagnosing sacroiliitis must be used, such as 99mTc-MDP single-photon emission CT (SPECT)/CT. Contraindications include patients with metal implants, pacemakers, and those who suffer from claustrophobia. In the study conducted by Parghane et al., planar imaging, bone SPECT/CT, and MRI were used for the diagnosis of sacroiliitis. The results of scans with SPECT/CT had greater sensitivity, diagnostic accuracy, and positive predictive value in comparison to planar bone scanning. Further, MRI was found to be superior when compared to planar bone scanning, scintigraphy, and radiography. Planar bone scanning had a sensitivity of 42.0%, while SPECT/CT was found to be 90.0%. The 99mTc-MDP SPECT had a higher sensitivity than planar scintigraphy; however, MRI was still found to be the most sensitive technique. The CT component of SPECT/CT has an advantage as it can evaluate erosions, subchondral sclerosis, joint space alternations, and new changes of bone that can help make the final diagnosis of sacroiliitis [2]. A consideration for greater accuracy in detecting SpA sacroiliitis is combining 99mTc-MDP SPECT with SPECT/CT to significantly increase the accuracy of the diagnosis.

The study conducted by Nadeem et al. consisted of patients over the age of 18 at a tertiary care center who had an SIJ MRI and then an SIJ CT completed within 12 months, with 14 weeks between the CT and MRI. The results concluded that SIJ CT has greater diagnostic confidence in relation to SIJ MRI [8]. Further, of the patients that had equivocal findings on MRI, 73.2% had a definitive diagnosis on CT, which demonstrates the increased confidence in detecting the presence of erosions on CTs [8]. Dual-energy CT in particular has the ability to visualize inflammation and structural changes as well as edema and subchondral bone marrow better than MRI, which can be used to evaluate the initial stages of SpA [8].

PsA

MRI: PsA is a distinct type of spondyloarthritis that presents with skin lesions as well as joint, bone, and cartilage abnormalities. PsA’s effects on the musculoskeletal system, including peripheral tendons and ligaments, make MRI a useful imaging modality for identifying patients with this SpA. Common findings on MRI for PsA include bony erosions, bone marrow and soft tissue edema, and tendon sheath effusions. In a study done by Ghanem et al., 25 patients with a clinical diagnosis of PsA were evaluated by two radiologists using T1-weighted, T2-weighted, and STIR imaging of the hands and feet [4]. All patients had at least one feature of PsA present on imaging [4]. Of the findings identified on MRI, bone marrow edema, bone erosion, soft-tissue edema, and joint effusion were visualized in 84%, 80%, 88%, and 92% of patients [4]. Contrast enhancement, depicting active lesions in the synovia, occurred in all 25 patients [4]. Similarly, in a separate study that utilized WB-MRI, synovitis of peripheral joints was more commonly identified than bone marrow edema and erosion [21]. Some joints showed equivalent synovitis when comparing PsA and SpA patients, such as the shoulder (48% vs. 42%) and the hips (both 17%), whereas other joints showed a preference for PsA [21]. A total of 48% of PsA knee joints depicted synovitis, compared to 17% of SpA patients. This study also found evidence of synovitis in 28%, bone marrow edema in 10%, and bone marrow erosions in only 1% of MCP joints in PsA patients [21]. A separate study found 85% of MCP joints to be abnormal on MRI imaging [4]. When looking at the joints of the hands and feet, 50% of patients with foot involvement had irregular imaging [4]. When compared to SpA, PsA had similar imaging findings in the hands and feet, whereas synovitis was more common in the feet for patients with SpA [21]. Dactylitis is one common finding in PsA and can be identified by looking at soft tissue edema and tendon sheath effusion on MRI [4]. Although peripheral joints such as the MCP and proximal interphalangeal (PIP) joints are often involved in PsA, WB-MRI had reduced visual acuity for these areas when compared to more proximal joints [21]. For instance, bone marrow edema readability in the knees was between 94% and 100%, but only 10% of DIPs were readable in comparison. Therefore, the knee was one of the best joints to view on MRI because it is more commonly inflamed in PsA than both the control group of participants and the participants with SpA. It is also more easily visualized on WB-MRI. While WB-MRI can be potentially utilized in PsA, small joints are not as easily depicted and can be missed on imaging [21].

US: Although MRI can identify peripheral manifestations of PsA, US has also been used. One study compared the clinical presentation of PsA with B-mode and power Doppler (PD) sonography. The Disease Activity Index for Psoriatic Arthritis (DAPSA) scores are based on tender joints, swollen joints, CRP levels, and patient input of disease activity and pain. Gray-scale US is one radiographic way to analyze synovitis in joints, and grading is based on joint capsule distension. PD US can also be used to identify inflammation in joints by visualizing the intra-article color signal or blood flow. In the study by Husic et al., a positive correlation between DAPSA and gray-scale synovitis (GSS) and PD joint scores was seen. Higher scores were found on USs for individuals with increased joint activity. The inflammatory marker, erythrocyte sedimentation rate, was also shown to have a weak-to-moderate correlation with GSS and PD joint US findings. Although overall scores showed moderate positive correlations to clinical presentation, enthesitis, dactylitis, and tenosynovitis were not associated individually with DAPSA. Patients with dactylitis seen on US were more likely to have active disease according to the Composite Psoriatic Disease Activity Index, a scoring system based on components of arthritis, skin manifestations, enthesitis, and dactylitis. Of the patients with US-verified dactylitis, a PD signal was seen in at least one tendon or joint. All patients had evidence of enthesophytes seen on US, and entheseal erosions were seen clinically in 67.1% of patients. While US findings did correlate with clinical signs of synovitis, its inability to accurately depict other peripheral lesions associated with PsA shows that it is a less reliable modality when compared to its MRI counterpart [24].

AS

MRI: AS is an inflammatory, seronegative disorder that typically affects the spine and larger joints of the body. MRI is the gold standard modality used to investigate the manifestations of AS. One of the early signs of AS is often sacroiliitis. Different MRI sequences, including fat-saturated and T2-weighted imaging, STIR, DWI, and contrast-enhanced imaging, can identify abnormalities in the SIJs. In one study of 18 individuals with moderate to severe AS with SIJ involvement, 17 were found to have abnormalities on imaging [15]. Common findings included bone marrow edema, erosion, subchondral fatty marrow infiltration, and irregular enhancement in the SIJs [15]. DWI is an MRI technique recently gaining popularity for identifying SI abnormalities due to its rapid and sensitive nature when compared to other MRI techniques that require contrast [25,26]. When comparing contrast to noise ratio, STIR had the highest image quality, but DWI and contrast-enhanced images had no significant difference [26]. Therefore, DWI may pose an interesting alternative to contrast imaging when visualizing sacroiliitis in early AS [26].

MRI can identify inflammation in the SIJs and in the vertebral body [27,28]. T1-weighted fat-saturated contrast-enhanced (Gd-DTPA) and STIR MRI sequences showed high reliability when visualizing inflammatory lesions in the spine in AS patients. Concordance amongst scoring in both MRI techniques and for both readers was found to be 83%. Both STIR and Gd-DTPA imaging showed high intraclass correlation when evaluating the entire spine for inflammatory lesions and showed low variance [28]. Evaluation of individual regions of the spine alone showed lower reliability and higher variance when compared to reviewing the entire spine [28]. Therefore, when identifying early AS, it is best to visualize the entire spine as opposed to the cervical, thoracic, and lumbar regions alone. The results of a separate study that used WB-MRI and STIR sequences to identify AS found two or more CILs in the spine to be 69% sensitive and 94% specific for AS [29]. Lateral inflammatory lesions had a high specificity of 97% but a low sensitivity compared to corner lesions, at only 31%. The concordance of this study among three radiologists was also high, at 80-85% [29]. WB-DWI images demonstrated hyperintense lesions in subchondral bone marrow in the spine, hip, ischial tuberosity, and pubic symphysis [26]. These extra-SI lesions correlated with clinical findings in patients with AS [26]. These findings emphasize not only the SIJ findings commonly associated with AS but also bring attention to how subchondral bone marrow abnormalities on WB-DWI may also be present in the spine and peripheral joints. The array of MRI sequences is often used to identify early AS because the array of sequences provides reliable and specific findings across several joints in the body associated with AS.

US: US can also be used to identify lesions associated with AS by detecting entheses and irregular vascularization seen in AS. One study found the US to be sensitive for identifying erosion, swelling, and new bone formation commonly seen with entheses but had low specificity when compared to plain radiographs [30]. When looking at overall enthesitis, US had 100% sensitivity for identifying erosion but only 20% specificity. When looking specifically at the quadricipital enthesis, new bone formation was both sensitive and specific, with values of 91.5% and 96% [30]. The Achilles tendon had an erosion sensitivity of 86% and a specificity of only 28% [30]. Specificity was based on comparing active lesions discovered in the US with those already found on radiographs. Therefore, low specificity can potentially be attributed to the lack of signal seen on plain radiographs in chronic enthesitis [30]. Sacroiliitis is a common early presentation of AS and, as mentioned previously, can be identified using color Doppler US (CDUS) [31]. One study found pulsatile monophasic flow on CDUS in 81.5% of patients with AS, and 18.5% had no flow [31]. This population underwent MRI as well, and 94.4% of individuals had positive findings [31]. This study found CDUS to have an 82% sensitivity and 92% specificity for identifying abnormal flow in patients with AS [31]. US and CT can also be utilized together to improve the overall image and get a better look at where abnormal vasculature occurs in the SIJ [32]. CT provides the added benefit of providing a higher-resolution image than the US and can provide specific anatomical information on irregular blood flow in patients with AS [32]. The utilization of CDUS to identify active sacroiliitis in patients with AS is a faster alternative to the gold standard MRI alternative. CT/US fusion imaging may improve the image quality of CDUS and provide a better picture when identifying active lesions in the SIJ.

CT: CT has been utilized as another modality to evaluate the SIJ in AS. One study compared the efficiency of CT to plain radiographs in identifying AS. A total of 37 patients with probable or definite AS were enrolled, and radiographs were graded using a modified NY criteria. Although plain radiographs and CT were both effective at identifying AS, CT graded higher on 32.4% of the right SIJs and 24.3% of the left SIJs. This was seen primarily in low-grade 0-1 plain radiograph lesions. Plain radiographs showed a higher grade of sacroiliitis in 10.8% of patients on the right and 5.4% on the left. When using the NY criteria, 10 individuals did not meet the criteria on plain radiographs but did, in fact, meet the criteria on CT. CT identified bilateral sacroiliitis in 86.5% of the individuals who underwent imaging, compared to only 75.7% of individuals under plain radiographs. The sensitivity of CT compared to plain radiographs may be beneficial in identifying early SI lesions associated with AS and may offer a lower-cost alternative when compared to MRI imaging [33]. Table 1 summarizes the results of all the studies included.

Discussion

SpAs are a group of seronegative and inflammatory arthritis, most commonly involving the spine. There are many chronic rheumatic diseases that can spread, leading to arthritis in joints, enthesitis, and dactylitis [1]. There are many imaging modalities used to evaluate SpA: MRI, CT, SPECT/CT, PET/CT, US imaging, low-dose CT, and DWI.

Overall, it is clear that MRI allows for greater sensitivity and specificity in diagnosing early sacroiliitis in patients [10,14]. Patient morbidity may decrease due to earlier diagnosis, resulting in therapeutic interventions that may help improve quality of life. STIR sequences proved to have greater utility when compared to DWI [11]. The 2D MRI had greater sensitivity than 3D isotropic imaging while also providing excellent diagnostic utility in characterizing the SIJ anatomy [14,17]. Furthermore, MRI provides a clear and accurate characterization of sacroiliitis and can be used to monitor chronic inflammatory changes and inform patient treatment.

Although MRI is found to be the superior imaging technique, PET has the potential to be more sensitive in relation to MRI and CT for detecting early bone remodeling changes, early inflammation, and/or structural sacroiliitis changes [16,23]. In relation to conventional bone scintigraphy, PET/CT scans have greater sensitivity, greater bone uptake, rapid blood clearance, and a better ability to detect erosions, sclerosis, and ankylosis [5]. SPECT/CT is useful when there are contraindications for MRI use, such as metal implants, pacemakers, and patients who suffer from claustrophobia [2]. Overall, MRI is the imaging technique of choice, but PET/CT shows greater promise in visualizing inflammation and structural changes, which is useful in evaluating the initial stages of SpA.

PsA presents with skin lesions along with joint, bone, and/or cartilage abnormalities. MRI is the most useful imaging modality for PsA because PsA affects mainly tendons and ligaments. On the contrary, WB-MRI was found to have greater difficulty when evaluating peripheral joints such as the hands and feet, specifically the MCP and PIP joints in PsA [21]. Therefore, US may be useful in identifying peripheral manifestations; however, US has difficulty relating to clinical signs of enthesitis, dactylitis, and tenosynovitis [24]. Thus, MRI is the imaging modality of choice and still has a greater ability to identify overall lesions of PsA, including peripheral lesions.

For AS, MRI is the gold standard modality. DWI, STIR, and T1-/T2-weighted images can identify not only abnormalities in the SIJs but also in peripheral and axial joints. Sequences such as STIR have the ability to suppress signals from fat while enhancing signals from inflammation, which allows for easier identification of vertebral and SI lesions [15]. DWI, on the other hand, has gained popularity because it is a quick alternative sequence that does not require contrast and can identify abnormalities based on the apparent diffusion coefficient [26]. When using MRI to identify vertebral joint abnormalities, it is important to look at the entire spine versus a certain anatomical region due to the decreased reliability of focusing on only the cervical, thoracic, or lumbar regions [28]. The different types of MRI sequences used to identify AS provide more specific and sensitive information when compared to other imaging modalities and therefore continue to be the gold standard.

Although MRI is typically used when investigating axial and peripheral lesions associated with AS, it can be expensive. US provides a potentially cost-effective alternative to identifying AS. US can detect entheses, changes in vascular patterns, erosions, and swelling in AS [30]. Although MRI is the imaging modality of choice in patients with AS due to its greater sensitivity and specificity, US, specifically Doppler US, can be a faster alternative. Further, CT is another imaging modality of choice for evaluating AS. It has a greater ability to detect lower grades of sacroiliitis in comparison to plain radiographs; however, MRI has the greatest sensitivity when comparing all three modalities [33]. Therefore, the US is a faster alternative to identify early SI lesions at a lower cost when compared to MRI.

Limitations

The PRISMA diagram (Figure 1) details the data collection process based on the initial inclusion criteria for the literature review search. From the starting 1,131 articles, 29 articles were chosen for this review. The specific search criteria for the study resulted in articles that were only relevant to the topic at hand within the past 10 years to ensure the data described were the most current findings in the medical field. Further, excluding literature that was older than 10 years and only in the English language removed some possibly important studies. However, this allowed the review of the literature to be the most comprehensive in the current field of medicine. The limited search criteria and focus on only peer-reviewed articles may have caused a handful of pertinent literature to be removed; however, it allowed the scoping review to focus on the most current and more specific evaluation of SpA.

Further, when interpreting the results in the literature, there were a few limitations. The majority of the articles determined results from a small sample size, which makes the findings difficult to generalize to a larger population. Secondly, a few of the studies did not have a control group, so a realistic outcome on the degree to which the imaging modality at hand evaluated SpA is questionable. Further, in the study conducted by Bredella et al., MRI was used to determine if it could be a useful imaging modality to determine active and inactive disease [15]. In this study, disease activity could not fully be confirmed because there was an absence of pathological tissue to evaluate, which affected the results. The study conducted by Nadeem et al. determined if CT could improve the diagnosis of SIJ erosions when patients had equivocal MRI findings [8]. In this study, the patients were rheumatology-referred patients, so the findings may not be accurate in routinely referred patients. Additionally, SIJ erosions could have developed within the 14.4-week waiting period between the initial SIJ MRI and subsequent CT.

Future Studies

Future studies should focus on obtaining a larger sample size to replicate results that are similar in clinical practices. Many of the manuscripts reviewed did not have a control group in the study, which is an important consideration for future studies to implement to more accurately determine the findings of the imaging modality. It is evident that MRI is the superior imaging technique, and future studies should focus on the ability of MRI to evaluate the possibility of disease onset in a patient as well as the continued evaluation of the progression of the disease. When comparing multiple imaging modalities, greater control of environmental factors and lifestyle should be present to ensure additional SIJ erosions do not occur, which can affect the results.

## Conclusions

SpA is a common cause of low back pain due to inflammatory arthritis in the spine manifesting as PsA, AS, and sacroiliitis. PsA is accompanied by skin and nail changes, along with joint pain and inflammation; it mainly affects tendons, bursae, and ligaments, making MRI the most useful imaging modality of choice. Further, AS presents with sacroiliitis that can spread to other regions of the spine. The predominant symptom of AS is IBP. MRI is shown to be the imaging modality of choice in AS due to its greater sensitivity in comparison to CT and plain radiographs. Similarly, MRI has the greatest sensitivity and specificity for diagnosing early sacroiliitis in patients. However, PET CT is another option when focusing on detecting early inflammation and bone remodeling changes because it has a greater ability to detect erosions, sclerosis, and ankylosis. Future studies should focus on increasing sample sizes and having a control group to monitor the accuracy of the imaging findings.
